# Welfare in horse breeding

**DOI:** 10.1136/vr.102814

**Published:** 2015-04-25

**Authors:** M. L. H. Campbell, P. Sandøe

**Affiliations:** 1 Department of Production and Population Health, Royal Veterinary College, Hawkshead Lane, South Mymms, Herefordshire AL9 7TA, UK; 2 Department of Large Animal Sciences and Department of Food and Resource Economics, University of Copenhagen, Rolighedsvej 25, 1958 Frederiksberg C, Denmark

## Abstract

Welfare problems related to the way horses are bred, whether by coitus or by the application of artificial reproduction techniques (ARTs), have been given no discrete consideration within the academic literature. This paper reviews the existing knowledge base about welfare issues in horse breeding and identifies areas in which data is lacking. We suggest that all methods of horse breeding are associated with potential welfare problems, but also that the judicious use of ARTs can sometimes help to address those problems. We discuss how negative welfare effects could be identified and limited and how positive welfare effects associated with breeding might be maximised. Further studies are needed to establish an evidence base about how stressful or painful various breeding procedures are for the animals involved, and what the lifetime welfare implications of ARTs are for future animal generations.

WELFARE problems relating to the way horses are bred – for example, stress related to long distance transport of breeding stallions or negative side effects of the application of artificial reproduction techniques (ARTs) – have been the occasional subject of conference presentations (for example, Campbell 2012, 2013, Mills 2013). However, they are not discussed a great deal within the horse industry, and have been given no discrete consideration within the academic literature.

This paper aims to address that deficit by reviewing the existing knowledge base on welfare issues in horse breeding, and identifying areas in which data is lacking. We then go on to discuss how negative welfare effects associated with horse breeding could be better identified and limited. ‘Horse breeding’ is defined for the purposes of this article as the processes which lead up to conception, pregnancy and the management of stallions, broodmares and foals until weaning.

In this review, we do not address welfare issues in horses that arise from heritable conditions (reviewed by Bettley and others 2012), nor welfare issues related to selective breeding for traits that humans find desirable (for example, extremes of size). Nor do we address the welfare issues of horse abandonment and neglect, identified by various equine charities and in the media as being caused by overbreeding of horses (for example, World Horse Welfare 2013).

Further, we do not, for reasons of space, discuss welfare issues that arise from the management of breeding animals. Instead, starting with the premise that breeding is a legitimate use of horses by people, our focus is on the welfare issues associated with the ways in which horses are bred, whether via ‘natural cover’ (coitus) or ARTs.

In the context of this paper, we are interested in ‘welfare’ in both negative and positive senses. We are concerned with protecting animals from negative experiences such as pain, discomfort, fear and stress, and also with maximising positive welfare effects. Although attempts to identify positive welfare effects of breeding on horses (for example, whether a mare takes pleasure in raising a foal) run the risk of descending into conjecture, it is possible to associate the fulfilment of animals' needs for social interaction, or the ability to exhibit some other normal behaviours, with positive welfare.

Welfare and health are linked throughout veterinary medicine, and this is particularly true of breeding, where vaccination of breeding stock and their offspring and control of venereal diseases are important tools in safeguarding welfare. However, in this article, we shall focus on issues that are welfare insults, in the sense that they can directly cause pain, discomfort, fear, stress or other forms of reduced welfare to an animal or its offspring, and shall not consider welfare issues that might arise from infectious disease processes (although infectious disease can, of course, cause pain and distress).

## Literature search

A search of the literature was carried out using the terms: ‘horse breeding and welfare’; ‘broodmares and welfare’; ‘stallions and welfare’; ‘foals and welfare’; ‘welfare effects of assisted reproductive technologies’; ‘welfare effects of assisted reproductive technologies in horses’; ‘equine embryo transfer’; ‘equine artificial insemination’; and ‘equine assisted reproductive technologies’. The search for books and peer-reviewed papers was conducted using the Royal Veterinary College's SCOUT system, PubMed, CAB direct, Biomed Central journals, and the Web of Science. Additionally, ‘soft’ data sources were searched using the same terms.

### Technique-associated welfare problems

### Natural cover

In the wild, a harem stallion and its band of mares interact constantly, year round (McDonnell 2000). Equine courtship and copulatory behaviours, in common with those of most mammalian species, are complex (Chenoweth and others 2014), and are frequently initiated by the mare rather than by the stallion (Chenoweth and others 2014; McDonnell 2000). In domesticated situations, although some breeders allow stallions to run loose with mares at pasture, it is much more common for horses to be bred either ‘in-hand’ (that is, the stallion is led by a human), or using ARTs.

Internationally, the stud books responsible for registering Thoroughbred horses will only do so for the purposes of racing if the horse has been conceived by ‘natural cover’, that is, by intercourse between a mare and stallion. Thoroughbreds conceived by artificial techniques, for example, artificial insemination (AI) or embryo transfer (ET), may not be raced under these rules. They may, however, be registered in an auxiliary part of the studbook and compete in disciplines other than racing, for example, eventing or polo. Mares being covered under a typical Thoroughbred stud farm system are restrained using a bridle and sometimes also the application of a nose twitch and hobbles, and have boots applied to the back feet to avoid them kicking the stallion. The stallion is typically led towards the rear of the mare, and expected to mount her with little if any opportunity to interact with her head end. This is very different from the natural behaviour of breeding horses, in which the mare frequently solicits the stallion, the initial approach from the stallion is often to the mare's head rather than her hindquarters, and an elaborate process of licking, vocalising and (sometimes) trial mounting is undertaken before copulation occurs (McDonnell 2000). In controlled, in-hand breeding the emphasis is on restraint of both horses. While the rationale behind such restraint is an understandable desire to protect both horses and humans from injury, it is arguable that there is the possibility of experienced frustration caused by preventing the animals from fulfilling behavioural needs (see also the fourth of the ‘five freedoms’, requiring the freedom to ‘express normal behaviour’ [FAWC 2012a]). No paper was found in the literature assessing the effect in terms of frustration or stress of a ‘natural cover’ system of this type on mares, foals or stallions.

Further welfare concerns associated with the use of ‘natural cover’ in the Thoroughbred industry relate to practices that are designed to maximise the economic benefit of breeding. A successful Thoroughbred stallion may cover up to 200 mares in a breeding season, which requires him to ‘perform’ two or three times a day, since the season is artificially limited by the studbooks (February 1 to July 15 in the Northern Hemisphere). Such heavy breeding schedules are an acknowledged cause of loss of libido (McDonnell 2011), and as such may indicate mental or physical stress. The practice of ‘shuttling’ Thoroughbred stallions between Northern and Southern Hemispheres so that they can be used for breeding activities year-round has the potential to further compromise their welfare by subjecting them to the stress of long flights, novel surroundings and unfamiliar pathogens.

### Time limitations

The artificial regulatory time limitation on the Thoroughbred breeding season also carries welfare implications. Naturally, mares are seasonal breeders. In the Northern Hemisphere, they would typically cycle regularly between about March – April and September. Under the rules of racing, all Thoroughbreds are given a nominal birth date – January 1 in the Northern Hemisphere – and race according to that nominal age. Because Thoroughbreds typically race from age two upwards (depending on the type of race) and many races are classified by age, a foal born in early January will have a competitive advantage over a foal born in June, which must race in the same age group. There is thus substantial economic pressure for mares to foal early. This requires mares to conceive at a stage of the year at which the majority of barren or maiden mares would naturally still be in seasonal anoestrus. Consequently, the use of artificial lighting, high planes of nutrition and drugs to stimulate reproductive cyclicity outside of the physiological breeding season in mares is commonplace (Sharp 2011). Where veterinarians and breeders are successful in stimulating mares to conceive early in the year, the resultant early-born foal may be confined indoors due to inclement weather and may experience a lack of grass when turned out; both challenges to the expression of normal behaviours (FAWC 2012a).

Many of the welfare concerns associated with the practice of natural cover could be alleviated or abolished by the use of AI. This allows for semen to be collected from a stallion, divided into aliquots and either frozen, delivered chilled or used fresh. Since one ejaculate typically contains enough semen to achieve pregnancies in four to seven mares, all but the busiest stallions need to be collected from only once a day at most. Because semen can be shipped internationally, there is no requirement for the stallion to travel. Typically a ‘dummy mare’ is used to collect the semen. Although this does not abolish the risk of injury to the stallion completely, it does mean that there is no risk of injury between a stallion and a real mare. Furthermore, when the semen can be shipped to the mare owner, mares and foals no longer need to be transported to stud, which reduces stress and exposure to pathogens.

### Assisted reproductive techniques

The vast majority of non-Thoroughbred studbooks now allow the use of ARTs. Although some non-Thoroughbred breeders still opt to use the kind of in-hand ‘natural cover’ system described above, breeding using AI and/or ET has become the norm for many breeds.

The use of ARTs, however, is not without its own welfare implications (Table 1). In considering the use of ARTs, we should be aware of short term and longer term welfare issues that affect animals either subjected to or derived from ARTs (Young and others 1998, Farin and others 2004, Farin and others 2006, Campbell and others 2014, Kim and others 2014).

**TABLE 1:** Summary of different procedures used in modern equine breeding, frequency of use, and the pros and cons of each technique from a welfare point of view

Technique	Frequency of use	Negative welfare effects	Positive welfare effects
Artificial insemination	Very common	Lack of controlled studies. Anecdotally likely to be minimally painful/stressful for most mares. Attenuation of normal reproductive behaviours for stallions. Anecdotally no evidence of long-term welfare effects on animals conceived by artificial insemination.	Reduces number of times/day stallion has to ejaculate. Reduces risk to stallion, mare and personnel during breeding. Abolishes need to transport stallions internationally and to transport mares and foals to stud. Ability to freeze semen facilitates castration of males, which makes it easier to manage social interaction between them and other animals.
Embryo transfer	Common in some countries, very common in others	Lack of controlled studies. Increased need for invasive examination and pharmacological manipulation compared with artifical insemination. Embryo flushing process may be stressful/painful.	Facilitates breeding from mares who would be at high risk of injury (for example, ventral rupture) if they carried the foal themselves. Possibility of shipping preserved embryos reduces the need to transport mares and foals.
Oocyte retrieval and transfer	Uncommon	Lack of controlled studies in mares. Known to be associated with increased heart rate and peripheral cortisol levels and development of adhesions in other species. No conclusive evidence of long-term welfare effects on foals conceived by oocyte retrieval, although these are known to occur in other species in association with particular uses of culture media.	
Cloning	Rare	Increased risk of abnormalities in foals at birth, increased requirement for neonatal intensive care.	

For stallions, potential welfare issues relate to what might be described as an attenuation of the freedom to express normal behaviours. The use of a ‘dummy mare’ minimises the risk of injury to the stallion without requiring restraint of a live mare, but also means that many successful breeding stallions in AI programmes never actually touch a mare or contact urine, faeces or urovaginal secretions of mares (McDonnell 2000). Such attenuation of normal behaviours is perhaps reflected in the fact that low libido in comparison to that seen in harem stallions is a recognised problem in stallions whose breeding behaviour is controlled by people (McDonnell 2000).

However, for the most part, potential welfare implications of the use of ARTs in equine breeding apply to mares or offspring, rather than to stallions. The most commonly used ARTs in equine breeding are AI and ET (Hartman 2011). Oocyte collection, gamete intrafallopian transfer (GIFT), in-vitro fertilisation (IVF), intracytoplasmic sperm injection (ICSI) and nuclear transfer (‘cloning’) are also viable techniques, although their application is less common in general practice than in specialised reproduction laboratories. A detailed discussion of the technical aspects, relative success and merits of equine ART procedures is outside the scope of this paper, but can be found in McKinnon and others (2011) and is reviewed by Hinrichs (2012).

With the exception of cloning (see below), little attention has been paid to the welfare implications of equine ARTs. However, techniques such as AI, ET and even GIFT are much more commonly undertaken than cloning. Therefore welfare issues associated with these techniques, if they exist, potentially affect a significant number of animals across the globe each year.

Mares can be inseminated with freshly collected, chilled or frozen semen, using simple transcervical, deep intrauterine or hysteroscopic insemination. It is recognised good practice to restrain a mare in stocks during these procedures, both for the safety of the personnel involved and to reduce the risk of a rectal tear occurring if the mare moves suddenly. Whether a mare is additionally sedated varies, and seems to depend upon the temperament of the mare and the perceived difficulty of the procedure. The majority of mares who are restrained in stocks and inseminated with fresh or chilled semen are not sedated. It is not common practice for mares to be provided with analgesia during AI (although where sedatives are used, some sedatives do have an analgesic component). No studies on pain and other negative states related to AI in mares were found in the literature.

### Embryo transfer

Similarly, there seem to be no studies on whether ET is painful in mares. Although ET is known to be painful in other species, especially those in which embryo flushing is a surgical procedure (Jirkof and others 2013), the fact that flushing and transfer are both usually now non-surgical procedures in mares and that the mare's cervix dilates easily even in dioestrus makes it likely that ET is a comparatively unpainful experience for mares. Nonetheless, it is common practice to sedate mares both during flushing and ET, but not to provide analgesia other than that incorporated in sedation. The rationale for such sedation is probably to reduce the risk of rectal tears and to make the procedure technically easier by preventing the mare moving. Hartman (2011) also suggested (apparently from clinical experience) that sedation is necessary during embryo flushing as fluid expansion of the uterus can be uncomfortable for the mare.

It is certainly true that the potential welfare issues for a donor mare, including those associated with the flushing procedure and with repeat injections to attempt to induce superovulation when used (Meyers-Brown and others 2010), exceed the potential welfare issues for the recipient mare (in whom non-surgical transfer differs little from a conventional AI). Overall, ET has greater potential to cause welfare issues than AI. Often, more than one recipient mare is prepared per donor mare, to increase the chances of achieving synchronisation of donor and recipient. This means that the number of invasive rectal and ultrasound examinations per pregnancy is increased compared to AI. Particularly where recipient mare numbers are limited, greater pharmacological manipulation (often involving repeated injection) of mares may also be necessary to achieve synchronisation than is necessary to manipulate the reproductive cycle for AI.

Embryo transfer can offer some positive welfare benefits insofar as the possibility of shipping preserved embryos abolishes the need to transport mares and foals. Flushing an embryo from a mare at risk of a pregnancy-related injury, for example, increased risk of a ventral musculature rupture during pregnancy due to previous colic surgery, also has the potential to improve that individual's welfare by abolishing the need for it to carry a foal to term.

### Oocyte retrieval and transfer

Oocyte retrieval (Carnevale 2011b) provides a method of achieving pregnancies in mares in which reproductive pathologies render ET unsuccessful (Galli and others 2001, Carnevale 2011a). In mares, unlike cattle, IVF has proved relatively unsuccessful (Hinrichs 2005). Oocyte retrieval is therefore more commonly used in conjunction either with transfer of the oocyte into a recipient mares' oviduct and simultaneous deposition of sperm in the oviduct (GIFT), or transfer of the oocyte into a recipient mare's oviduct followed by transcervical insemination of the recipient. Alternatively, the oocyte can be fertilised in vitro using ICSI, and the embryo created is placed in the recipient mare's uterus using ET.

Although various techniques for oocyte retrieval have been described (Carnevale 2011a), the most commonly used is transvaginal ultrasound-guided follicular aspiration (Cook and others 1993, Galli and others 2001, Carnevale 2011a). Transvaginal ultrasound-guided follicular aspiration in women is known to be associated with pain, the severity of which is dependent upon needle design (Wikland and others 2011). The insertion of a needle through the vaginal wall carries associated risks of pathogen transmission and of vaginal rupture which are assumed to apply also in animals (McEvoy and others 2006). In sheep and goats, repeated transvaginal oocyte retrieval has been associated with the development of adhesions (McEvoy and others 2006). In cattle, one study associated transvaginal ultrasound-guided follicular aspiration with an increase in heart rate and peripheral cortisol levels, although the authors commented that those effects may have been in reaction to epidural anaesthesia rather than to oocyte puncture specifically (Petyim and others 2007). Another study failed to demonstrate any significant difference in alteration of cortisol levels or milk production between cows subjected to repeated ovum pickup and control animals (Chastant-Maillard and others 2003).

Perhaps because the number of mares having oocyte retrieval performed on them is much lower than the number of farm animals being subjected to the same ART, we know less about welfare compromising side effects in mares. Although the effect of repeated aspiration of follicles on fertility has been studied (Mari and others 2005), the specific question of whether oocyte retrieval in mares causes pain or discomfort does not seem to have been addressed. Cook and others (1993), although not aiming to investigate whether oocyte retrieval caused pain, did incidentally report that ovaries appeared to become painful after aspiration of several follicles, and that one of 30 mares showed signs of pain after aspiration. Despite the lack of specific studies on whether oocyte aspiration causes pain in the mare, it is nonetheless normal practice for mares undergoing oocyte retrieval to be restrained in stocks, sedated and provided with systemic analgesia (Cook and others 1993, Galli and others 2001, Carnevale and others 2003, Carnevale 2011a). The perceived need to restrain the mare may be related to concerns about causing rectal tears and about the ease of follicular aspiration, as well as about pain. Similar provision is made for mares into which an oocyte is being surgically transferred, in combination with local anaesthetic (Carnevale 2011b). The need for analgesia during transfer is perhaps more obvious since an incision is being made. Recipient mares also have to undergo aspiration of their own preovulatory follicle(s) in order to ensure that the oocyte that is fertilised following transfer originates from the donor and not the recipient mare. Neither surgical transfer nor aspiration of the recipient's own follicle is necessary if oocyte retrieval is used in conjunction with ICSI and a non-surgical ET rather than GIFT.

Relatively little evidence is available about the effects of oocyte aspiration on foals created using retrieved oocytes. In cattle, where oocyte retrieval is often used in combination with IVF and ET, that combination has been associated with fetal oversize, compromised placental competence, and perinatal deaths due to cardiovascular, pulmonary or other limitations (McEvoy and others 2006). The welfare problems encountered with bovine oocyte retrieval followed by IVF and ET may relate to technique and the length of time and conditions under which oocytes and embryos are held in vitro (McEvoy and others 2006). Fetal oversize in particular has been shown to be related to culture conditions, including the addition of serum to embryo culture media (Everts and others 2008, Smith and others 2009, Angulo and others 2010). It does not, therefore, necessarily follow that similar problems would occur in mares undergoing oocyte retrieval and GIFT, or ICSI and ET. Indeed, where information about foals conceived using these methods is available in the literature, there is no evidence that these techniques have negative effects on the health and other aspects of the welfare of foals, at least in the short term.

### Cloning

Recent publications about the efficiency of producing ‘clones’ by equine somatic cell nuclear transfer, and discussion among stud books and regulatory authorities about the registration of clones and their offspring (FEI 2012), have promoted consideration of the welfare implications associated with this particular equine ART. Current cloning techniques result in recognised welfare problems in farm animals (Renard and others 2001, Houdebine and others 2008, FAWC 2012b). Problems begin during the embryo stages, when loss rates are higher than rates associated with other ARTs (EFSA 2010), and continue right through to the adult stages of the lives of cloned animals (Renard and others 2001).

In equine reproduction, production of embryos using somatic cells as the source of nuclear transfer is now both a research tool and a clinical service (Hinrichs 2005, 2006, 2012). Because the number of cloned foals is still small (estimated at 100 to 200 worldwide [Hinrichs 2012]), and perhaps because there is not the same public concern about the possible effects on human health (EFSA 2008), data about the health and welfare of equine clones is sparse compared to that available for clones of species that are primarily used for food production. What little data is available comes primarily from the efforts of the group led by Katrin Hinrichs at Texas A&M University to collate information relating to the production of ‘cloned’ foals (Hinrichs 2006, Johnson and others 2010). This group reported that 26 per cent of cloned embryos transferred by them resulted in the birth of a live foal. This is a higher success rate than that of Galli and coworkers, who produced three live foals from transfers of more than 100 cloned embryos (Galli and others 2003, Lagutina and others 2005).

Although only dealing with the data from one laboratory, the study by Johnson and others (2010) suggests that the incidence of abnormalities at birth in ‘cloned’ foals exceeds that of non-cloned foals, and that cloned foals require intensive treatment if they are to survive the immediate postpartum period. Umbilical abnormalities (as in other species) seem a particular problem. However, the fetal oversize and consequent dystocia seen in cattle seem to not occur in horses. This is probably because mares generally ‘regulate’ the size of their foal in utero (Allen and others 2002). Similarly, the problems of hydrops of the fetal membranes, which occur in cloned cattle, seem not to occur in mares; this may be due to differences in placentation.

## Limiting negative welfare effects

It is clear from this review that known and potential welfare issues are associated with the use of horses for breeding, whether the technique used be ‘natural’ or ‘artificial’.

There is a strong argument to be made that some types of ART are useful tools for improving the welfare of breeding horses. For example, use of AI and shipped semen removes the need to transport mares, foals and stallions nationally or internationally, and thus reduces their exposure to stress and infectious disease. Transportation of flushed chilled embryos rather than a whole mare has similarly positive welfare implications. A recent innovation by SportHorse Breeding (UK) whereby the studbook has agreed to allow geldings with previously frozen semen to be entered into the stallion grading scheme and, if they pass, to be registered as breeding animals, is another good example of using ARTs to improve welfare. The ability to use geldings as breeding animals will remove the need to keep male animals entire, and should therefore make it easier to keep them in a system where welfare-enhancing social interaction with other horses is easier to manage. Additionally, knowing that geldings have the potential to be registered as breeding animals later in life might encourage owners to have semen frozen before the animal is gelded, and this in turn might reduce the incentive to clone a gelding after he has proven competitively successful in order to use his clone as a breeding animal. Given the welfare issues associated with current cloning techniques this too should be welfare-enhancing.

However, notwithstanding the potential of some ARTs to improve equine welfare, more information is needed about possible negative welfare effects of equine ARTs, particularly the newer ones. A lack of information about the safety and efficacy of new ARTs is not unique to animal reproduction and has also been identified in the field of human reproductive medicine (Dondorp and de Wert 2011). The current situation in equine reproduction seems to be a somewhat paradoxical one whereby analgesia is provided for some techniques (for example, oocyte retrieval) in the absence of an evidence base proving that the technique is painful, and not provided for other techniques (for example, AI) in the absence of an evidence base proving that they are not painful. Clinical experience suggests that most veterinarians are making decisions about the need to provide analgesia based on observations of the mare's behaviour and supposition about what might be painful. Generally, clinicians seem to err on the side of providing analgesia if they suspect that the mare might experience discomfort.

The ability of owners and veterinarians to make evidence-based judgements about the negative welfare effects of different types of ARTs and about the potential effect of using analgesia requires studies incorporating valid measures of pain, discomfort, fear, stress and other aspects of negative welfare in the mares involved. Such studies could include measurement of physiological parameters such as salivary or serum cortisol levels in horses before and after an ART is used (Peeters and others 2011). However, there are good reasons to be critical of physiological measures as stand-alone welfare measures, and to try instead to combine physiological measurements with behavioural indicators (Robertson and Sanchez 2010). Horses, like other prey species, tend to mask or minimise signs of pain and other welfare problems. Behavioural indicators are often subtle (Ashley and others 2005). Examples of such subtle indicators are activity level, level of contact with peers and humans (Pader and others 2011), and facial expression (Dalla Costa and others 2014, Gleerup and others 2014). It is possible to define numerical rating scales for behavioural and other indicators of pain in horses (Bussiéres and others 2008, Robertson and Sanchez 2010, van Loon and others 2014), and it would, for the purpose of assessing the welfare effects of ARTs, be relevant to develop specific genital/gynaecological pain scales for horses.

Further information is also needed about the effect of ARTs on the welfare of future generations of horses. AI and ET are techniques that have been used on mares for decades now. Hinrichs (2012) estimates that 25,000 ETs are performed worldwide per year. Common-sense suggests that if long-term welfare problems in offspring created using AI and ET were an issue, they would have become anecdotally obvious and been reported in the literature by now. It therefore seems likely that long-term welfare effects on future animals do not occur in association with AI and ET. However, there is a paucity of information about the long-term effects of more recently developed ART techniques, such as ICSI and cloning, on the welfare of future generations. Multicentre, long-term cohort studies of horses created using such techniques would inform decision making about the use and modification of techniques.

## Conclusions

There is a general lack of data about the welfare of breeding horses, which could be rectified by the addition of such horses as a separate category to ongoing data-gathering exercises about general horse welfare. Careful attention to the management of breeding horses in ways which enable them to express normal behaviours as fully as possible within the constraints of needing to avoid injury to horses and people would improve welfare, both by reducing negative effects and by increasing positive, welfare-maximising factors. Breeders and veterinarians should also give careful thought to whether insults to welfare arising from recognised stressors could be reduced by employing ARTs. However, further studies are needed in order to establish an evidence base about how stressful/painful various ARTs are for the animals involved, and what the lifetime welfare implications of ARTs are for future animal generations. Only when that information becomes available will we be able to make sound ethical judgements about whether the (potential) cost of (possibly) stressful/painful techniques is outweighed by a welfare benefit derived from reduced exposure to stress associated with travel, mixing of animals, and ‘natural’ cover techniques.

## References

[R1] ALLEN W., WILSHER S., TURNBULL C., STEWART F., OUSEY J., ROSSDALE P., FOWDEN A. (2002) Influence of maternal size on placental, fetal and postnatal growth in the horse. Development in utero. Reproduction 123, 445–453 11882022

[R2] ANGULO J., GENTRY G. T., GODKE R. A., BONDIOLI K. R (2010) Effect of serum during culture on day 14 elongated bovine embryos. Reproduction, Fertility and Development 23, 191

[R3] ASHLEY F. H., WATERMAN-PEARSON A. E., WHAY H. R. (2005) Behavioural assessment of pain in horses and donkeys: application to clinical practice and future studies. Equine Veterinary Journal 37, 565–575 16295937 10.2746/042516405775314826

[R4] BETTLEY C. D., CARDWELL J. M., COLLINS L. M., ASHER L. (2012) A review of scientific literature on inherited disorders in domestic horse breeds. Animal Welfare 21, 59–64

[R5] BUSSIÉRES G., JACQUES C., LAINAY O., BEAUCHAMP G., LEBLOND A., CADORE J-L., DESMAIZIERES L-M., CUVELLIEZ S. G., TRONCY E. (2008) Development of a composite orthopaedic pain scale in horses. Research in Veterinary Science 85, 294–306 18061637 10.1016/j.rvsc.2007.10.011

[R6] CAMPBELL M. L. H (2012) The ethics of equine cloning. BEVA Congress, Birmingham www.myeventflo.com/event.asp?part=3&evID=1324&lectID=300. Accessed April 20, 2015

[R7] CAMPBELL M. L. H (2013) Pushing the boundaries in competition and breeding horses – is there an alternative to abolition? nAWSELVA Conference, London

[R8] CAMPBELL M. L. H, MELLOR D. J., SANDØE P. E (2014) How should the welfare of fetal and neurologically immature post-natal animals be protected? Animal Welfare 23, 369–379 26973382 10.7120/09627286.23.4.369 PMC4786996

[R9] CARNEVALE E. M. (2011a) Mature oocyte collection. In Equine Reproduction. Eds McKinnon A. O., Squires E. L., Vaala W. E., Varner D. D. Wiley Blackwell pp 2936–2939

[R10] CARNEVALE E. M. (2011b) Oocyte transfer. In Equine Reproduction. Eds McKinnon A. O., Squires E. L., Vaala W. E., Varner D. D. Wiley Blackwell pp 2941–2944

[R11] CARNEVALE E. M., COUTINHO DA SILVA M. A., SQUIRES E. L. (2003) How to collect and transfer oocytes. In 49th Annual Convention of the American Association of Equine Practitioners www.ivis.org/proceedings/AAEP/2003/carnevale/chapter_frm.asp?LA=1. Accessed July 10, 2014

[R12] CHASTANT-MAILLARD S., QUINTON H., LAUFFENBURGER J., CORDONNIER-LEFORT N., RICHARD C., MARCHAL J., MORMEDE P., RENARD J. P. (2003) Consequences of transvaginal follicular puncture on well-being in cows. Reproduction 125, 555–563 12683926 10.1530/rep.0.1250555

[R13] CHENOWETH P. J., LANDAETA-HERNÁNDEZ A. J., FLÖERCKE C. (2014) Reproductive and maternal behavior of livestock. In Genetics and Behaviour of Domestic Animals. Eds Grandin T., Teesing M. J. Elsevier pp 160–183

[R14] COOK N. L., SQUIRES E. L., RAY B. S., JASKO D. J. (1993) Transvaginal uItrasound-guided follicular aspiration of equine oocytes. Equine Veterinary Journal Supplement 15, 71–74

[R15] DALLA COSTA E., MINERO M., LEBELT D., STUCKE D., CANALI E., LEACH M. C. (2014) Development of the horse grimace scale (HGS) as a pain assessment tool in horses undergoing routine castration. PLOS One 9, e92281 10.1371/journal.pone.0092281 PMC3960217 24647606

[R16] DONDORP W., DE WERT G. (2011) Innovative reproductive technologies: risks and responsibilities. Human Reproduction 26, 1604–1608 21502181 10.1093/humrep/der112

[R17] EFSA (2008) Outcome of Public Consultation on the EFSA Draft Animal Cloning Opinion. www.efsa.europa.eu/en/supporting/pub/834.htm. Accessed April 20, 2015

[R18] EFSA (2010) Update on the state of play of animal cloning. EFSA Journal 8, 1784

[R19] EVERTS R. E., CHAVATTE-PALMER P., RAZZAK A., HUE I., GREEN C. A., OLIVEIRA R., VIGNON X., OTHERS (2008) Aberrant gene expression patterns in placentomes are associated with phenotypically normal and abnormal cattle cloned by somatic cell nuclear transfer. Physiological Genomics 33, 65–77 18089771 10.1152/physiolgenomics.00223.2007

[R20] FARIN C. E., FARIN P. W., PIEDRAHITA J. A. (2004) Development of fetuses from in vitro-produced and cloned bovine embryos. Journal of Animal Science 82, Suppl E53–62 15471815 10.2527/2004.8213_supplE53x

[R21] FARIN P. W., PIEDRAHITA J. A., FARIN C. E. (2006) Errors in development of fetuses and placentas from in vitro-produced bovine embryos. Theriogenology 65, 178–191 16266745 10.1016/j.theriogenology.2005.09.022

[R22] FAWC (2012a) The five freedoms. http://webarchive.nationalarchives.gov.uk/20121007104210/http://www.fawc.org.uk/freedoms.htm. Accessed April 20, 2015

[R23] FAWC (2012b) Welfare implications of cloning: letter to Lord Taylor. www.gov.uk/government/uploads/system/uploads/attachment_data/file/324771/FAWC_advice_on_the_cloning_of_farm_animals.pdf. Accessed April 20, 2015

[R24] FEI (2012) Sports forum: cloning. www.fei.org/system/files/VET_CLONING.pdf. Accessed April 20, 2015

[R25] GALLI C., CROTTI G., NOTARI C., TURINI P., DUCHI R., LAZZARI G. (2001) Embryo production by ovum pick up from live donors. Theriogenology 55, 1341–1357 11327688 10.1016/s0093-691x(01)00486-1

[R26] GALLI C., LAGUTINA I., CROTTI G., COLLEONI S., TURINI P., PONDERATO N., DUCHI R., LAZZARI G. (2003) A cloned horse born to its dam twin. Nature 424, 635 12904778 10.1038/424635a

[R27] GLEERUP K. B., FORKMAN B., LINDERGAARD C., ANDERSEN P. H. (2014) An equine pain face. Veterinary Anaesthesia and Analgesia doi: 10.1111/vaa.12212 10.1111/vaa.12212 PMC4312484 25082060

[R28] HARTMAN D. L. (2011) Embryo Transfer. In Equine Reproduction. Eds McKinnon A. O., Squires E. L., Vaala W. E., Varner D. D. Wily Blackwell pp 2871–2879

[R29] HINRICHS K. (2005) Update on equine ICSI and cloning. Theriogenology 64, 535–541 15985289 10.1016/j.theriogenology.2005.05.010

[R30] HINRICHS K. (2006) Equine cloning. Veterinary Clinics of North America: Equine Practice 22, 857–866 17129808 10.1016/j.cveq.2006.07.004

[R31] HINRICHS K. (2012) Assisted reproduction techniques in the horse. Reproduction, Fertility and Development 25, 80–93 10.1071/RD12263 23244831

[R32] HOUDEBINE L-M., DINNYÉS A., BÁNÁTI D., KLEINER J., CARLANDER D. (2008) Animal cloning for food: epigenetics, health, welfare and food safety aspects. Trends in Food Science and Technology 19 Suppl v1, S88–S95

[R33] JIRKOF P., FLEISCHMANN T., CESAROVIC N., RETTICH A., VOGEL J., ARRAS M. (2013) Assessment of postsurgical distress and pain in laboratory mice by nest complexity scoring. Laboratory Animal 47, 153–161 10.1177/0023677213475603 23563122

[R34] JOHNSON A. K., CLARK-PRICE S. C., CHOI Y-H., HARTMAN D. L., HINRICHS K. (2010) Physical and clinicopathologic findings in foals derived by use of somatic cell nuclear transfer: 14 cases (2004-2008). Journal of the American Veterinary Medical Association 236, 983–988 20433399 10.2460/javma.236.9.983

[R35] KIM M. J., OH H. J., KIM G. A., JO Y. K., CHOI J., KIM H. J., CHOI H. Y., KIM H. W., CHOI M. C., LEE B. C. (2014) Reduced birth weight, cleft palate and preputial abnormalities in a cloned dog. Acta Veterinaria Scandinavica 56, 18 24669802 10.1186/1751-0147-56-18 PMC3984017

[R36] LAGUTINA I., LAZZARI G., DUCHI R., COLLEONI S., PONDERATO N., TURINI P., CROTTI G., GALLI C. (2005) Somatic cell nuclear transfer in horses: effect of oocyte morphology, embryo reconstruction method and donor cell type. Reproduction 130, 559–567 16183874 10.1530/rep.1.00772

[R37] MARI G., MERLO B., IACONO E., BELLUZZI S. (2005) Fertility in the mare after repeated transvaginal ultrasound-guided aspirations. Animal Reproduction Science 88, 299–308 16143219 10.1016/j.anireprosci.2005.01.002

[R38] MCDONNELL S. M. (2000) Reproductive behavior of stallions and mares: comparison of free-running and domestic in-hand breeding. Animal Reproduction Science 60, 211–219 10844196 10.1016/s0378-4320(00)00136-6

[R39] MCDONNELL S (2011) Abnormal sexual behaviour. In Equine Reproduction. Eds McKinnon A. O., Squires E. L., Vaala W. E., Varner D. D. Wiley Blackwell pp 1407–1412

[R40] MCEVOY T. G., ALINK F. M., MOREIRA V. C., WATT R. G., POWELL K. A. (2006) Embryo technologies and animal health – consequences for the animal following ovum pick-up, in vitro embryo production and somatic cell nuclear transfer. Theriogenology 65, 926–942 16280157 10.1016/j.theriogenology.2005.09.008

[R41] MCKINNON A. O., SQUIRES E. L., VAALA W. E., VARNER D. D. (2011) Equine Reproduction. Wiley Blackwell

[R42] MILLS G. (2013) How far can we push the animals we use? Veterinary Record 172, 518–519 23687203 10.1136/vr.f3143

[R43] MEYERS-BROWN G. A., MCCUE P. M., NISWENDER K. D., SQUIRES E. L., DELUCA C. A., DIDSTRUP L. A., COLGIN M., FAMULA T. R., ROSER J. F. (2010) Superovulation in mares using recombinant equine follicle stimulating hormone: ovulation rates,embryo retrieval, and hormone profiles. Journal of Equine Veterinary Science 30, 560–568

[R44] PADER K., FREEMAN L. J., CONSTABLE P. D., WU C. C., SNYDER P. W., LESCUN T. B. (2011) Comparison of transvaginal natural orifice transluminal endoscopic surgery (NOTESR) and laparoscopy for elective bilateral ovariectomy in standing mares. Veterinary Surgery 40, 998–1008 22091993 10.1111/j.1532-950X.2011.00877.x

[R45] PEETERS M., SULON J., BECKERS J. F., LEDOUX D., VANDENHEEDE M. (2011) Comparison between blood serum and salivary cortisol concentrations in horses using an adrenocorticotropic hormone challenge. Equine Veterinary Journal 43, 487–493 21496072 10.1111/j.2042-3306.2010.00294.x

[R46] PETYIM S., BAGE R., MADEJ A., LARSSON B. (2007) Ovum pick-up in dairy heifers: does it affect animal well-being? Reproduction in Domestic Animals 42, 623–632 17976070 10.1111/j.1439-0531.2006.00833.x

[R47] RENARD J. P., QI ZHOU R., LEBOURHIS D., CHAVATTE-PALMER P., HUE I., HEYMAN Y., VIGNON X. (2001) Nuclear transfer technologies: between successes and doubts. Theriogenology 57, 203–222 11775970 10.1016/s0093-691x(01)00667-7

[R48] ROBERTSON S. A., SANCHEZ L. C. (2010) Treatment of visceral pain in horses. Veterinary Clinics of North America Equine Practice 26, 603–617 21056302 10.1016/j.cveq.2010.08.002

[R49] SHARP D. C. (2011) Vernal transition into the breeding season. In Equine Reproduction. Eds McKinnon A. O., Squires E. L., Vaala W. E., Varner D. D. Wiley Blackwell pp 1704–1715

[R50] SMITH S. L., EVERTS R. E., SUNG L. Y., DU F., PAGE R. L., HENDERSON B., RODRIGUEZ-ZAS S. L., OTHERS (2009) Gene expression profiling of single bovine embryos uncovers significant effects of in vitro maturation, fertilization and culture. Molecular reproduction and Development 76, 38–47 18449896 10.1002/mrd.20927

[R51] VAN LOON J. P. A. M., JONCKHEER-SHEEHY V. S. M., BACK W., VAN WEEREN P. R., HELLEBREKERS L. J (2014) Monitoring equine visceral pain with a composite pain scale score and correlation with survival after emergency gastrointestinal surgery. Veterinary Journal 200, 109–115 10.1016/j.tvjl.2014.01.003 24491373

[R52] WIKLAND M., BLAD S., BUNGUM L., HILLENSJO T., KARLSTROM P. O., NILSSON S. (2011) A randomized controlled study comparing pain experience between a newly designed needle with a thin tip and a standard needle for oocyte aspiration. Human Reproduction 26, 1377–1383 21467200 10.1093/humrep/der100 PMC3096561

[R53] WORLD HORSE WELFARE (2014) www.worldhorsewelfare.org/Article/Charity-rescues-nearly-60-horses-from-excessive-breeder. Accessed April 20, 2015

[R54] YOUNG L. E., SINCLAIR K. D., WILMUT I. (1998) Large offspring syndrome in cattle and sheep. Reviews of Reproduction 3, 155–163 9829550 10.1530/ror.0.0030155

